# Großes Makulaforamen – immer eine schlechte Prognose?

**DOI:** 10.1007/s00347-020-01178-3

**Published:** 2020-07-14

**Authors:** J. Friedrich, N. Bleidißel, J. Klaas, N. Feucht, A. Nasseri, C. P. Lohmann, M. Maier

**Affiliations:** 1 Klinikum rechts der Isar, Augenklinik, Technische Universität München, Ismaninger Str. 22, 81675 München, Deutschland; 2 Smile Eyes Augenklinik Munich Airport, Terminalstraße Mitte 18, 85356 München, Deutschland

**Keywords:** iSD-OCT, Intraoperative Bildgebung, Hochpräzisionsvitreoretinalchirurgie, Makulachirurgie, SD-OCT, iSD-OCT, Surgical imaging, Vitreoretinal surgery, Macular surgery, SD-OCT

## Abstract

**Hintergrund:**

Alter, präoperativer Visus und Makulaforamengröße gelten als prognostische Marker für das postoperative Ergebnis bei Patienten mit durchgreifendem Makulaforamen (MF).

**Ziel der Arbeit:**

Um den postoperativen Verlauf nach i‑ILM Peeling (inverted-Flap ILM-Peeling) mit konventionellem ILM-Peeling (k-ILM) zu vergleichen, wurde eine retrospektive Beobachtungsstudie durchgeführt. Patienten mit i‑ILM Peeling hatten dabei präoperativ ein statistisch signifikant größeres Makulaforamen.

**Material und Methoden:**

Es wurden 45 konsekutive Patienten mit durchgreifendem Makulaforamen (MF) in 2 Gruppen (i-ILM vs. k‑ILM) eingeteilt und auf Unterschiede im postoperativen Visus (BCVA) und der Netzhautmorphologie hin untersucht. Die Integrität der äußeren Netzhautschichten, äußere limitierende Membran (ELM), ellipsoide Zone (EZ) und äußere Photorezeptoraußensegmente (OS), wurde postoperativ mittels SD-OCT (Spectral-Domain-OCT) analysiert.

**Ergebnisse:**

Die präoperative Apertur in der i‑ILM Gruppe war signifikant größer (i-ILM = 408,4 µm, SD = 157,5 µm; k‑ILM = 287,4 µm, SD = 104,9 µm; *p* = 0,01). Der Ausgangsvisus sowie der postoperative Visus nach 1 Monat waren in der Gruppe mit k‑ILM-Peeling signifikant besser (*p* = 0,03 und *p* = 0,001). Der postoperative Visus nach mindestens 6 Monaten zeigte keinen signifikanten Unterschied zwischen den beiden Gruppen (*p* = 0,24). Die ELM zeigte als erste der äußeren Netzhautschichten eine Re-Integrität in beiden Gruppen.

**Schlussfolgerung:**

Mithilfe der i‑ILM-Peeling-Technik erschien es in dieser konsekutiven Serie möglich zu sein, für Patienten mit großem durchgreifendem MF ein ähnliches postoperatives Visusergebnis zur erreichen wie für mittels k‑ILM-Peeling-Technik operierte Patienten mit kleinerem durchgreifendem MF.

## Abstract (en)

**Background:**

Age, preoperative visual acuity and macular hole size have been reported to be prognostic markers for the postoperative outcome in patients with full thickness macular holes (FTMH).

**Objective:**

A retrospective observational clinical study was performed in order to compare the postoperative course after conventional internal limiting membrane peeling (c-ILM) and inverted flap ILM peeling (i-ILM). Patients with i‑ILM peeling had preoperatively a significantly larger macular hole

**Material and methods:**

In this study 45 consecutive patients with a full thickness macular hole (FTMH) were divided into two groups (c-ILM vs. i ILM) and evaluated with respect to the postoperative best corrected visual acuity (BCVA) and retinal morphology. Using spectral domain optical coherence tomography (SD-OCT) the integrity of the outer retinal layers, external limiting membrane (ELM), ellipsoid zone (EZ) and outer photoreceptor outer segments (OS) were analyzed postoperatively for at least 6 months.

**Results:**

The preoperative aperture in the i‑ILM group was significantly larger (i-ILM = 408.4 µm, SD = 157.5 µm; c‑ILM = 287.4 µm, SD = 104.9 µm; *p* = 0.01). The preoperative BCVA and postoperative BCVA after 1 month were significantly better in the group with c‑ILM peeling (*p* = 0.03 and *p* = 0.001). The postoperative BCVA after at least 6 months showed no significant difference between the two groups (*p* = 0.24). The ELM was the first of the outer retinal layers to show postoperative recovery in both groups.

**Conclusion:**

Using the i‑ILM peeling technique in this consecutive series it seemed to be possible to achieve a similar postoperative visual outcome in patients with a large FTMH as for patients with a smaller FTMH operated on with the c‑ILM peeling technique.

## Hintergrund und Fragestellung

Die Spectral-Domain-OCT (SD-OCT) [6] ermöglicht eine Scangeschwindigkeit von bis zu 80.000 A-Scans pro Sekunde [7] und stellt damit derzeit den Goldstandard unter anderem in der Diagnostik von durchgreifendem Makulaforamen (MF) dar [9, 10].

Neben Alter und präoperativem Visus gilt die mittels SD-OCT erfassbare präoperative Makulaforamengröße als prognostischer Marker für das postoperative Ergebnis bei Patienten mit durchgreifendem Makulaforamen [8].

Dank intraoperativer OCT-Videoaufnahmen in Echtzeit [9] ist es dem Operateur möglich, mikrostrukturelle Veränderungen der Retina zu beobachten und die operative Strategie anzupassen. Neben der besseren Visualisierung unterschiedlicher Operationstechniken, wie beispielsweise der Inverted-Flap-ILM-Peeling-Technik (i-ILM), führt dies durch präzisere Durchführung [13] zu einer höheren Sicherheit in der vitreomakulären Chirurgie [5, 12, 18].

Eine retrospektive Beobachtungsstudie wurde durchgeführt, um den postoperativen Verlauf von Patienten, die mittels Inverted-ILM-Flap-Peeling (i-ILM) operiert wurden, mit dem postoperativen Verlauf jener Patienten zu vergleichen, die mit konventionellem Membranpeeling (k-ILM) operiert wurden. Patienten mit i‑ILM-Peeling hatten präoperativ ein statistisch signifikant größeres Makulaforamen.

Es besteht ein Zulassungsvotum der Ethikkommission der TU München.

## Studiendesign und Untersuchungsmethoden

**Figure 1:**
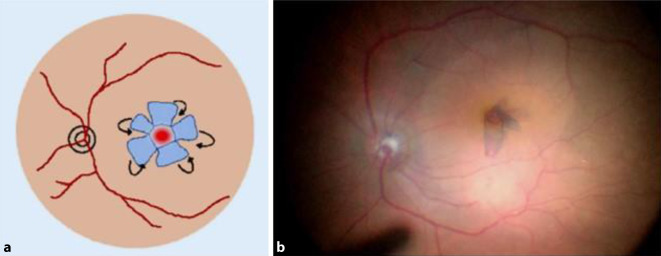

**Figure 2:**
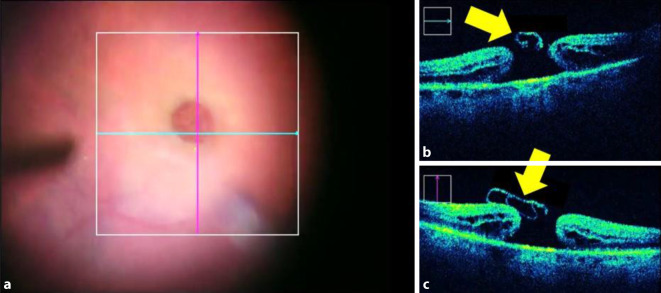

Es wurden 45 Augen von 45 konsekutiven Patienten mit durchgreifendem Makulaforamen untersucht, welche im Zeitraum Juni 2015 bis Dezember 2017 operiert worden waren. Als Einschlusskriterium galt ein idiopathisches, durchgreifendes Makulaforamen, wobei Komorbiditäten wie Netzhautablösung, Glaskörperblutung und jegliche zuvor durchgeführte Netzhautoperation sowie die medikamenteninduzierte Vitreolyse mittels Ocriplasmin als Ausschlusskriterium galten. Die Patienten wurden in 2 Gruppen unterteilt, je nachdem ob die Patienten mittels Inverted-ILM-Flap-Membranpeeling (i-ILM) (Apertur im Mittel = 408,4 µm) oder mittels konventionellen ILM-Membranpeelings (k-ILM) operiert wurden (Apertur im Mittel = 287,4 µm). Es erfolgte in beiden Gruppen eine standardisierte transkonjunktivale, nahtlose 23-Gauge-Pars-plana-Vitrektomie sowie iOCT- und Brilliant Blue G- (G-81005 Brilliant Peel©; Fluoron, Geuder AG, Heidelberg, Deutschland) assistiertes ILM-Peeling mit anschließendem Luft-Gas-Austausch (C3F8, 12 %). Bei Patienten mit großem Makulaforamen wurde beim ILM-Peeling mithilfe des iOCT zusätzlich ein ILM-Flap so präpariert, dass durch eine schmale Brücke am Foramenrand ein Scharnier entsteht, über welches der Flap mit der vitrealen Seite nach unten auf das Foramen gelegt wurde (Inverted-ILM-Flap) [11]. Das Vorgehen ist in Abb. 1 und in Abb. 2 veranschaulicht.

Die intraoperative Bildgebung mittels iSD-OCT erfolgte in beiden Gruppen nach einem standardisierten Scan-Protokoll zu Beginn der Operation, nach Induktion der hinteren Glaskörperabhebung, nach ILM-Peeling sowie am Ende der Operation und ist bei einem Patienten mit i‑ILM-Peeling beispielhaft in Abb. 2 dargestellt. Die Operation wurde jeweils vom selben Ophthalmochirurgen (M. M.) mithilfe des Lumera 700 OP-Mikroskop (Carl Zeiss Meditec AG, Jena, Deutschland) mit integriertem Rescan 700 intraoperativem OCT durchgeführt.

Präoperativ wurden Linsenstatus und Visus sowie die Foramengröße mittels SD-OCT erhoben. Die Foramengröße wurde gemessen an der Apertur, welche definiert ist als geringster Durchmesser des Makulaforamens parallel zum retinalen Pigmentepithel (RPE), gemessen auf Höhe der mittleren Netzhautschichten [4]. Die Auswertung der Foramengröße erfolgte anhand der Software „Heidelberg Eye Explorer – Heyex“ (Software Serial-Nr.: H2E-18404-028-011, Heidelberg Engineering GmbH, Heidelberg, Deutschland).

**Figure 3:**
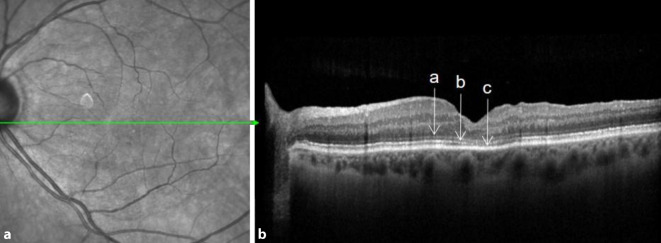

Postoperativ wurden 2 Kontrollen durchgeführt. Im Intervall von 3 bis 12 Wochen postoperativ fand die erste Kontrolle statt, nach mindestens 6 Monaten erfolgte die zweite postoperative Kontrolle. Es wurden für jeden Patienten Linsenstatus, Visus und Verschluss des Foramens erhoben. Die Morphologie der äußeren Netzhautschichten externe limitierende Membran (ELM), ellipsoide Zone (EZ) sowie Photorezeptoraußensegmente (OS) wurden mittels SD-OCT in einem Stern- oder Volumenscan erfasst und auf Intaktheit hin analysiert. Die äußeren Netzhautschichten galten dann als „intakt“, wenn in keiner der vorliegenden Scan-Linien ein Defekt der jeweiligen Netzhautschicht erkennbar war. Die postoperative Bildgebung mittels SD-OCT ist beispielhaft in Abb. 3 dargestellt.

Die statistische Auswertung der Daten erfolgte mithilfe der Software IBM SPSS Statistics Version 22 (IBM, New York, USA). Das Signifikanzniveau wurde für einen *p*-Wert *p* = 0,05 festgelegt. Prä- und postoperative Daten wurden mittels deskriptiver Statistiken analysiert. Der postoperative Visus wurde mithilfe des t‑Tests für verbundene Stichproben mit dem präoperativen Visus verglichen.

## Ergebnisse

Von 45 Patienten waren 35 Patienten (78 %) weiblich und 10 Patienten (22 %) männlich. Somit ergibt sich ein Verhältnis von weiblichen zu männlichen Patienten von näherungsweise 4:1. Das durchschnittliche Alter betrug 67 Jahre (SD = 8,5 Jahre, Minimum = 38 Jahre, Maximum = 85 Jahre).

Die i‑ILM-Gruppe fasste 30 Patienten, in der k‑ILM-Gruppe waren es 15 Patienten. In der i‑ILM-Gruppe betrug das durchschnittliche Alter 65 Jahre, in der k‑ILM-Gruppe betrug das durchschnittliche Alter 72 Jahre (*p* = 0,02).

Die mittlere Größe des Makulaforamens, gemessen an der Apertur, betrug in der i‑ILM-Gruppe 408,4 µm (SD = 157,5 µm) und in der k‑ILM-Gruppe 287,4 µm (SD = 104,9 µm). Die i‑ILM-Gruppe umfasste somit Makulaforamina mit einer signifikant größeren Apertur (*p* = 0,01).

Der mittlere bestkorrigierte Visus betrug präoperativ in der i‑ILM-Gruppe 0,81 logMAR (SD = 0,35 logMAR) und in der k‑ILM-Gruppe 0,58 logMAR (SD = 0,26 logMAR). In der i‑ILM-Gruppe waren 5 Patienten (17 %) präoperativ pseudophak, in der k‑ILM-Gruppe waren es 6 Patienten (40 %). Präoperativ zeigte sich somit ein signifikant schlechterer Visus in der i‑ILM-Gruppe, also bei Patienten mit großem Makulaforamen (*p* = 0,03).

Der bestkorrigierte Visus lag zum Zeitpunkt der ersten postoperativen Kontrolle in der i‑ILM-Gruppe im Mittel bei 0,49 logMAR (SD = 0,29 logMAR) und in der k‑ILM-Gruppe im Mittel bei 0,21 logMAR (SD = 0,11 logMAR). Der postoperative Visus verbesserte sich somit zum Zeitpunkt der ersten postoperativen Kontrolle um 0,32 logMAR für Patienten der i‑ILM-Gruppe und um 0,37 logMAR für Patienten der k‑ILM-Gruppe. Bei der ersten postoperativen Kontrolle, 3 Wochen bis 3 Monate postoperativ, zeigte sich demnach der Visus in der i‑ILM-Gruppe ebenfalls noch signifikant schlechter als in der k‑ILM-Gruppe (*p* < 0,01).

**Figure 4:**
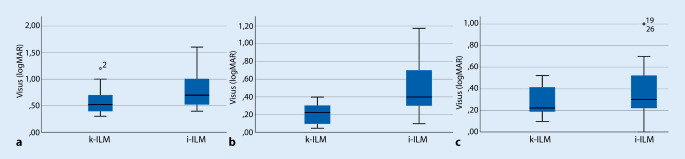

Der Mittelwert für den bestkorrigierten Visus bei der zweiten postoperativen Kontrolle betrug in der i‑ILM-Gruppe 0,40 logMAR (SD = 0,26 logMAR) und 0,28 logMAR (SD = 0,16 logMAR) in der k‑ILM-Gruppe. Zum Zeitpunkt der zweiten postoperativen Kontrolle verbesserte sich der Visus somit um 0,41 logMAR für Patienten der i‑ILM sowie um 0,3 logMAR für Patienten der k‑ILM-Gruppe im Vergleich zum Ausgangsvisus. Zum Zeitpunkt der zweiten postoperativen Kontrolle, mindestens 6 Monate postoperativ, zeigte sich somit in den beiden Gruppen kein signifikanter Unterschied mehr (*p* = 0,24). Die Gegenüberstellung der Visuswerte ist in Abb. 4 veranschaulicht.

**Figure 5:**
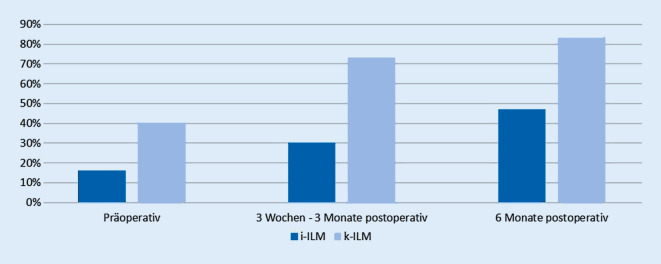

Präoperativ betrug die Pseudophakierate in der i‑ILM-Gruppe 16 %, in der k‑ILM-Gruppe betrug die Pseudophakierate 40 %. Zum Zeitpunkt der ersten postoperativen Kontrolle hatten 70 % der i‑ILM-Gruppe und 27 % der k‑ILM-Gruppe noch keine Kataraktoperation. Zum Zeitpunkt der zweiten postoperativen Kontrolle waren 53 % der i‑ILM-Gruppe und 17 % der k‑ILM-Gruppe noch phak. Die Pseudophakierate der beiden Gruppen ist in Abb. 5 dargestellt.

Die Evaluation der postoperativen Netzhautmorphologie ergab, dass die externe limitierende Membran (ELM) die erste der äußeren Netzhautschichten war, welche eine Re-Integrität zeigte. Bei der ersten postoperativen Kontrolle zeigte sich bei 75 % der Patienten eine intakte ELM, allerdings nur bei 22 % der Patienten eine intakte ellipsoide Zone (EZ) und bei 29 % intakte Photorezeptoraußensegmente (OS). Bei der zweiten postoperativen Kontrolle zeigten sich bereits bei der Hälfte aller Patienten auch die äußeren Netzhautschichten ellipsoide Zone (50 %) und Photorezeptoraußensegmente (53 %) intakt.

Der Zusammenhang zwischen Integrität der äußeren Netzhautschichten und postoperativem Visus wurde ebenfalls untersucht.

Der postoperative Visus nach 3 Wochen bis 3 Monaten betrug bei Patienten mit intakter ELM im Mittel 0,34 logMAR (SD = 0,23 logMAR), bei Patienten mit defekter ELM betrug er im Mittel 0,55 logMAR (SD = 0,36 logMAR). Bei Patienten mit intakter EZ betrug der postoperative Visus nach 3 Wochen bis 3 Monaten im Mittel 0,46 logMAR (SD = 0,22 logMAR), bei defekter EZ im Mittel 0,39 logMAR (SD = 0,30 logMAR). Bei Patienten mit intakter OS betrug der postoperative Visus nach 3 Wochen bis 3 Monaten im Mittel 0,44 logMAR (SD = 0,25 logMAR), bei defekter EZ im Mittel 0,38 logMAR (SD = 0,28 logMAR).

Der postoperative Visus nach 6 Monaten betrug bei Patienten mit intakter ELM im Mittel 0,31 logMAR (SD = 0,18 logMAR), bei Patienten mit defekter ELM betrug er im Mittel 0,60 logMAR (SD = 0,30 logMAR). Es zeigte sich somit in der Gruppe von Patienten mit intakter ELM ein signifikant besserer postoperativer Visus als bei Patienten mit unterbrochener ELM (*p* = 0,02) zum Zeitpunkt der zweiten postoperativen Kontrolle. Ein ähnliches Ergebnis konnte bei Patienten mit intakter EZ und bei Patienten mit intakter OS zum Zeitpunkt der zweiten postoperativen Kontrolle festgestellt werden (jeweils *p* < 0,01). Bei intakter EZ ebenso wie bei intakter OS betrug der postoperative Visus nach 6 Monaten im Mittel 0,26 logMAR (SD = 0,14 logMAR), bei defekter EZ bzw. defekter OS dagegen im Mittel 0,50 logMAR (SD = 0,28 logMAR).

**Figure 6:**
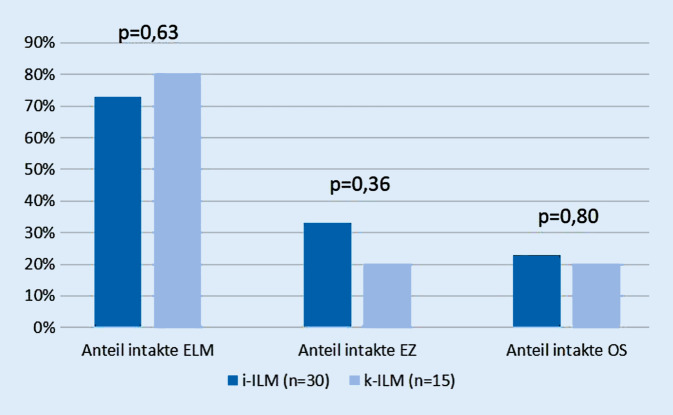

Beim Vergleich der beiden Gruppen, k‑ILM und i‑ILM, zeigte sich kein statistisch signifikanter Unterschied bezogen auf die Integrität der äußeren Netzhautschichten (Abb. 6 und 7). Nach 3 Wochen bis 3 Monaten postoperativ zeigte sich die ELM in der i‑ILM-Gruppe in 73 % und in der k‑ILM-Gruppe in 80 % intakt (*p* = 0,63). Die EZ erschien in der i‑ILM-Gruppe in 33 % und in der k‑ILM in 20 % intakt (*p* = 0,36), die OS war in der i‑ILM-Gruppe in 23 % und in der k‑ILM-Gruppe in 20 % intakt (*p* = 0,80).

**Figure 7:**
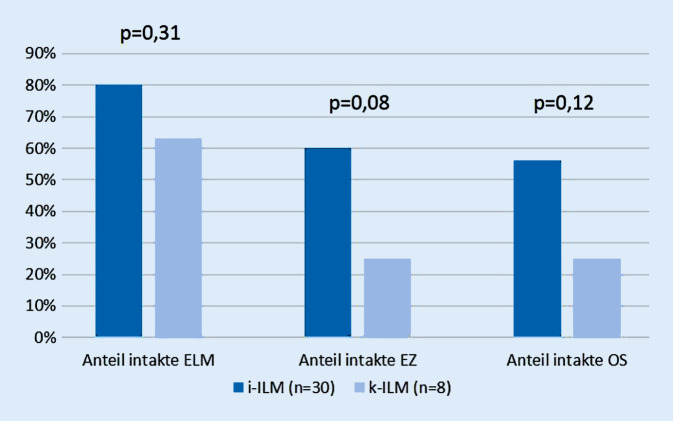

Nach mindestens 6 Monaten zeigte sich die ELM in der i‑ILM-Gruppe in 80 % und in der k‑ILM-Gruppe in 63 % intakt (*p* = 0,31). Die EZ erschien in der i‑ILM-Gruppe in 60 % und in der k‑ILM in 25 % intakt (*p* = 0,08), die OS war in der i‑ILM-Gruppe in 56 % und in der k‑ILM-Gruppe in 25 % intakt (*p* = 0,12). Aufgrund mangelnder Datensätze konnten in der Analyse der äußeren Netzhautschichten zum Zeitpunkt der zweiten postoperativen Kontrolle in der k‑ILM-Gruppe nur *n* = 8 Patienten untersucht werden.

Zum Zeitpunkt der ersten postoperativen Kontrolle, nach 3 Monaten, zeigte sich im SD-OCT bei allen 45 Patienten (100 %) ein verschlossenes Makulaforamen.

## Diskussion

Das i‑ILM-Peeling konnte bereits in zahlreichen Studien als wirksame, sichere Operationstechnik identifiziert werden [11, 13, 15, 17].

Neuere Studien weisen darauf hin, dass die Regeneration der äußeren Netzhautschichten, insbesondere der ELM, bei mittels i‑ILM-Peeling operierten Patienten früher eintritt.

Die Heilung der Netzhaut beginnt nach erfolgreicher Makulaforamenchirurgie im Bereich der inneren Netzhautschichten und weitet sich über die ELM zu den äußeren Netzhautschichten aus [3]. Die Regeneration der ELM scheint somit eine Voraussetzung für die Regeneration weiterer äußerer Netzhautschichten, v. a. der EZ zu sein [1, 2, 14]. Eine intakte Schicht von Photorezeptoraußensegmenten wiederum bedingt einen besseren postoperativen Visus, was den verzögerten Visusanstieg nach erfolgreichem Makulaforamenschluss erklärt [3].

**Figure 8:**
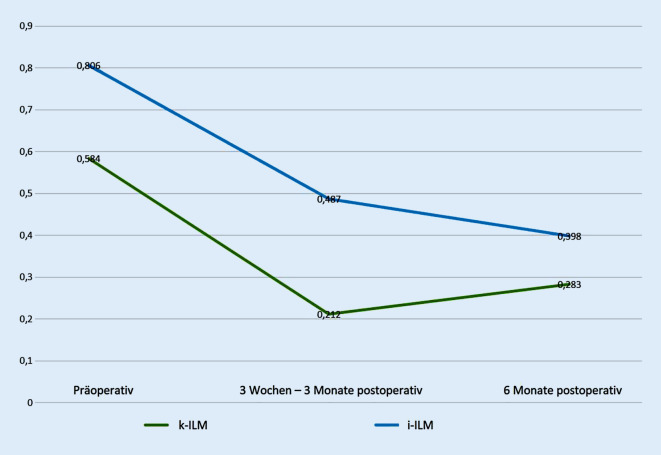

Der postoperative Visus scheint bei i‑ILM-Patienten kurzfristig schneller anzusteigen, im Langzeitverlauf scheint es jedoch keinen Visusunterschied zwischen Patienten, die mittels i‑ILM-Peeling operiert wurden, und Patienten, die mittels k‑ILM-Peeling operiert wurden, zu geben [1, 16]. Diese Beobachtung konnte in der vorliegenden Arbeit dahingehend gestützt werden, dass sich der Visusunterschied zwischen den beiden Gruppen i‑ILM und k‑ILM im Beobachtungszeitraum verringerte, wie in Abb. 8 verdeutlicht.

Bei einer Verschlussrate von 100 % in beiden Gruppen kann in dieser Arbeit die These, dass das i‑ILM-Peeling zu einer höheren Verschlussrate führt, nicht bestätigt werden.

Eine Limitierung dieser Arbeit ist, dass die Dauer der Symptomatik bis zur Operation nicht erfasst wurde und keine genauere Analyse des präoperativen vitreoretinalen Übergangs z. B. im Hinblick auf präoperative vitreomakuläre Traktionen erfolgte. Das postoperative Visusergebnis ist vermutlich negativ von der natürlichen Kataraktentwicklung der Patienten beeinflusst, da es sich um ein Patientenkollektiv mit einem mittleren Alter von 67 Jahren handelt. Eine Subgruppenanalyse der pseudophaken Patienten erfolgte nicht.

Zusammenfassend weisen unsere Ergebnisse darauf hin, dass ein großes durchgreifendes Makulaforamen nicht unbedingt ein schlechtes Visusergebnis vorhersagt. Der postoperative Visus von Patienten mit einem großen Makulaforamen, welche mittels i‑ILM-Peeling operiert wurden, nähert sich nach 6 Monaten dem postoperativen Visus von Patienten mit kleinerem Makulaforamen, die nicht mittels i‑ILM-Peeling operiert wurden, an. Das i‑ILM-Peeling scheint damit eine vielversprechende Therapie für Patienten mit großem Makulaforamen zu sein. Um diese These weiter zu stützen, sind weitere Untersuchungen erforderlich, welche Patientengruppen mit der gleichen Foramengröße und unterschiedlichen Operationstechniken vergleichen.

## Fazit für die Praxis

- Wenn Patienten mit großem MF mit i‑ILM-Peeling operiert werden, haben sie einen vergleichbaren Outcome wie Patienten mit kleinem MF.
- Die ELM zeigt als Erste der äußeren Netzhautschichten eine postoperative Regeneration.
- Die ELM scheint eine Voraussetzung für die Heilung der anderen äußeren Netzhautschichten zu bilden.
- Der postoperative Visus ist vom Heilungsstatus der äußeren Netzhautschichten abhängig.
